# Microsatellite markers for an endemic Atlantic Forest tree, *Manilkara multifida* (Sapotaceae)

**DOI:** 10.1093/aobpla/plt006

**Published:** 2013-01-28

**Authors:** Ramiris C. S. Moraes, Caio V. Vivas, Fernanda A. Oliveira, Ivandilson P. P. Menezes, Cassio van den Berg, Fernanda A. Gaiotto

**Affiliations:** 1 Centro de Biotecnologia e Genética, Universidade Estadual de Santa Cruz (UESC), Rodovia Ilhéus-Itabuna km 16s/n, Salobrinho, 45662-900 Ilhéus, Bahia, Brazil; 2 Departamento de Ciências Biológicas, Universidade Estadual de Feira de Santana (UEFS), Av. Transnordestina s/n, 44036-900 Feira de Santana, Bahia, Brazil

**Keywords:** Conservation, fragmentation, molecular ecology, molecular marker, PCR, population genetics, SSR, tropical rainforest.

## Abstract

*Manilkara multifida* is a tropical tree that is endemic to the Atlantic forests of southern Bahia, Brazil. Currently, populations of this species are restricted to fragmented landscapes that are susceptible to anthropogenic disturbances. Considering this issue, and that there is no genetic information available for this endangered species, we developed microsatellite markers for *M. multifida* to provide resources for future conservation genetics studies. Using an enriched genomic library, we isolated eight polymorphic microsatellite loci and optimized the amplification conditions for *M. multifida*. For each locus, we estimated the number of alleles, *H*_E_ and *H*_O_, paternity exclusion *Q*, individual identity *I* and fixation index *F*, and examined the presence of null alleles. The mean number of alleles was 11.9, and the heterozygosity was high at all loci (average *H*_E_ = 0.809 and *H*_O_ = 0.777). The combined values for both paternity exclusion and individual identity were *Q* = 0.9959 and *I* = 5.45 × 10^–11^, respectively. No evidence of null alleles was detected. The results of our analysis indicated that all eight microsatellites are promising for assessing questions involving inbreeding, gene flow, co-ancestry and mating patterns in *M. multifida*.

## Abstract (precis)

We developed microsatellites for *Manilkara multifida* for future conservation genetics studies. *M. multifida* is a tropical tree that is endemic to Brazil which is currently restricted to fragmented landscapes. Our analysis indicated that all eight microsatellites are promising for assessing population genetics questions in this species.

## Introduction

The Atlantic Forest is the second largest tropical forest in South America. Its original area measured ∼1 500 000 km^2^ ranging along the Brazilian Atlantic coast, with additional patches in Argentina and Paraguay (Ribeiro *et al.* 2009). Currently, only 11.4–16% of its original cover remains, and it is distributed in a fragmented landscape (Ribeiro *et al.* 2009) as a consequence of land use and occupation. Most Brazilians live in areas that were originally covered by the Atlantic Forest, which increases the pressure on native forest species. Southern Bahia, Brazil, is a particularly diverse region of the Atlantic Forest, especially in terms of tree species, and for many years, inadequate forest management and agriculture intensification reduced the amount of forest cover as well as the abundance of tree species. During the 1970s, the timber industry alone caused major environmental damage in this region (Mesquita 1997), and several species, including *Manilkara multifida* Penn., suffered a significant reduction in that period due to the high commercial value of their timber.

*Manilkara multifida* is a rare tree species endemic to the Atlantic Forest of southern Bahia, and it is included on the IUCN Red List as an endangered species (O'Brien 1998). Members of the genus *Manilkara* are known to provide important food resources for primates (Oliveira *et al.* 2009), but there are no data about the functional and evolutionary role of *M. multifida* in its habitat. Trees of this species are lactescent and have flowers with red sepals and white petals (Pennington 1990). They can also be identified by their wide and discolorous leaves, which are covered by an adpressed indumentum on the abaxial face, and by their numbers of petals, stamens and staminoids (8–9 altogether) (Pennington 1990). In the present study, we report the development and characterization of the first microsatellite markers for *M. multifida.* This resource will help researchers obtain experimental data for the elaboration of genetic inferences that will contribute to conservation programmes for this species.

## Methods

### Genomic library and primer design

A genomic library was constructed from template DNA (250 ng μL^−1^) extracted via the cetyl trimethylammonium bromide method (Doyle and Doyle 1990). Isolated DNA was digested with the restriction enzyme RsaI (Invitrogen, Carlsbad, CA, USA). Fragments of between 200 and 800 bp were captured and linked to the adapters Rsa21 and Rsa25 (Edwards *et al.* 1996) using T4 DNA ligase. The DNA fragments containing microsatellites were captured by hybridization with biotinylated probes (CT)_8_ and (GT)_8_, and recovered using magnetic streptavidin beads (Promega, Madison, WI, USA). These fragments were then amplified by polymerase chain reaction (PCR) according to the following conditions: initial denaturation at 95°C for 1 min; 25 cycles of denaturation at 94°C for 40 s, annealing at 60 °C for 40 s and extension at 72 °C for 2 min; and final extension at 72 °C for 5 min. Amplicons were cloned into the pGEM-T Easy vector (Promega), which was then transformed into *Escherichia coli* XL1-Blue (Promega). A set of 96 positive clones was amplified using M13 primers and Big Dye^®^ Terminator reagents (version 3.1; Applied Biosystems, Foster City, CA, USA) and sequenced using a DYEnamic^TM^ kit (GE Healthcare). Among these, 22 clones were discarded due to low-quality sequencing data (chromatograms). We analysed the remaining 74 sequences using SSRLocator software (Maia *et al.* 2008) and detected 67 containing microsatellites. A total of 14 pairs of primers were designed using Primer3 software (Rozen and Skaletsky 2000).

### Characterization of microsatellite loci

We tested the optimized primers in two steps: PCR amplification and electrophoretic analysis. The PCR reactions contained genomic DNA (7.5 ng), ultrapure water, 1× buffer (Fermentas), bovine serum albumin (2.5 ng µL^−1^), dNTPs (2.5 mM), MgCl_2_ (25 mM), tailed M13 forward and reverse primers (10 µM) labelled with DS-33 fluorescent dyes (NED^TM^, 6-FAM^TM^, PET^®^ or VIC^®^ dyes; Applied Biosystems) and *Taq* DNA polymerase (1 U). Reactions were carried out in a BioEr LifePro thermal cycler under the following conditions: 94 °C for 3.5 min, 35 cycles of 94 °C for 1 min, *T*_a_ °C for 45 s, 72 °C for 1 min and 72 °C for 15 min. Eight primer pairs successfully amplified DNA fragments from samples from 32 *M. multifida* individuals from the RPPN Estação Veracel (Porto Seguro, Bahia, Brazil) and Reserva Biológica de Una (Una, Bahia, Brazil) conservation units. The sizes of the labelled amplified fragments were determined by capillary electrophoresis (ABI 3130XL Genetic Analyzer) with the internal size standard GS500 LIZ (Applied Biosystems) using GeneMapper software (Applied Biosystems).

### Analyses performed

We assessed paternity exclusion *Q*, diversity indices *H*_E_ and *H*_O_, and allelic identity (*I*) using Cervus 3.0.3 (Marshall *et al.* 1998). We also computed the fixation index (*F*) in FSTAT 2.9.3.2 (Goudet 2002) and examined for null alleles using Micro-Checker (Van Oosterhout *et al.* 2004).

## Results

Eight polymorphic SSR loci for *M. multifida* were identified (Table 1). The average of 11.9 alleles per locus indicated high levels of polymorphism. The expected heterozygosity ranged from 0.622 to 0.911, and the observed heterozygosity ranged from 0.563 to 0.969. The MM03 and MM86 loci showed negative values of *F* (indicating excess heterozygotes). The combined exclusion probability of *Q* indicates that this set of microsatellites will be a powerful tool for future paternity studies. The *I* combined estimation confirms the potential use of this set of markers from *M. multifida* for identity analyses (Table 2). Null alleles were not observed at any locus, according to the Brookfield 1 method (Brookfield 1996). In many cases, the presence of null alleles, which usually occur due to a mutation in the primer annealing region, inflates the frequency of homozygotes. The Micro-Checker software found no genotype scoring errors due to stutter bands or allelic dropout.

**Table 1:** Description of the eight nuclear SSR markers developed for *M. multifida* in the present study, with forward (F) and reverse (R) sequences, repeat motif, annealing temperature and size range.

Locus	Primer sequence (5′–3′)	Repeat motif	*T*_a_ (°C)	Size range (bp)
MM03	F: CTGCATTGTAGCCGAAGAGA R: CAGGTGCAGCCAGCTCT	(AC)_7_n31(AG)_9_	52	202–242
MM07	F: CAGATTCGAGATAAGTGGAACTCA R: AGGCACATCCACATATGCAC	(TG)_10_n64(AT)_4_	56	172–202
MM56	F: TGAGTGCCGGACTCTCACT R: ATCATCTCCATGCGACACAC	(CA)_10_	54	294–324
MM62	F: CCAAACAGAGTGCAACTAAA R: CCCATCCCTATTAACAGTTG	(CT)_15_(CA)_7_	56	270–298
MM78	F: GGGTGAGCACATGGTCAAA R: CACACTGTTCAACTTCAATGGTATC	(ATAA)_6_	58	158–224
MM83	F: GCCCGACCCTCCTTATTAAA R: CCAAGATGTATGTTGGGATGAA	(TG)_6_n49(GT)_11_	58	330–362
MM86	F: AAGTAGTTGGCAATTCAACAGG R: ATATTGCCATTAACGCATCG	(GT)_7_n4(AT)_3_	52	194–218
MM90	F: TCAAGGTGCTTGATATCTGA R: CAGCCGTATTAATGGAGTGT	(TC)_4_n16(AC)_9_	52	262–294

**Table 2:** **Population genetic parameters calculated for eight microsatellite loci developed for *M. multifida*: number of alleles (Na), expected heterozygosity (*H*_E_), observed heterozygosity (*H*_O_), paternity exclusion index (*Q*), identity analysis (*I*), and fixation index (*F*)**.

Locus	Na	*H*_E_	*H*_O_	*Q*	*I*	*F*
MM03	13	0.828	0.969	0.4956	0.0480	−0.173
MM07	11	0.855	0.750	0.5266	0.0420	0.125
MM56	07	0.700	0.563	0.2806	0.1472	0.199
MM62	15	0.911	0.844	0.6563	0.0193	0.075
MM78	17	0.888	0.813	0.6076	0.0266	0.087
MM83	11	0.764	0.625	0.3832	0.0849	0.185
MM86	08	0.622	0.813	0.2176	0.1874	−0.313
MM90	13	0.903	0.844	0.6338	0.0224	0.067
Mean	11.9	0.809	0.777	0.9959^a^	5.45 × 10^−11b^	0.040

^a^Combined exclusion probability.

^b^Combined estimation.

## Conclusions and forward look

The eight polymorphic microsatellite markers developed in this study display important discrimination capacities. These loci therefore have excellent potential to be used in genetic studies of *M. multifida* aimed at the conservation of this species.

## Accession numbers

The sequences were deposited in the GenBank database with the following accession numbers: JX183540 [MM03]; JX183541 [MM07]; JX183542 [MM56]; JX183543 [MM62]; JX183544 [MM78]; JX183545 [MM83]; JX183546 [MM86]; JX183547 [MM90].

## Sources of funding

This work was funded by the Fundo de Amparo a Pesquisa da Bahia (FAPESB; PNX0014/2009 and APR0192/2008). R.C.S.M was supported by a fellowship from Coordenação de Aperfeiçoamento de Pessoal de Ensino Superior (CAPES), and F.A.G. was supported by a research fellowship from the Conselho Nacional de Desenvolvimento Científico e Tecnológico (CNPq).

## Contributions by the authors

R.C.S.M. collected samples in the field and participated in the primer design, characterization of the microsatellite loci and manuscript editing. C.V.V. participated in the fieldwork and data analysis. F.A.O. participated in the data analysis. I.P.P.M. developed the genomic library. C.v.d.B. participated in the microsatellite marker validation and manuscript editing. F.A.G. participated in the microsatellite marker validation and manuscript editing.

## Conflict of interest statement

None declared.
